# When the Patient Believes That the Organs Are Destroyed: Manifestation of Cotard's Syndrome

**DOI:** 10.1155/2016/5101357

**Published:** 2016-11-28

**Authors:** Leonardo Machado, Luiz Evandro de Lima Filho, Liliane Machado

**Affiliations:** ^1^Neuropsychiatry Department and Postgraduation in Neuropsychiatry and Behavior Sciences Program, The Federal University of Pernambuco, 50670-901 Recife, PE, Brazil; ^2^Psychology Department, The ESUDA College, 50050-100 Recife, PE, Brazil; ^3^Hospital of Mental Health Professor Frota Pinto, 60841-110 Fortaleza, CE, Brazil

## Abstract

Cotard's Syndrome (CS) is a rare clinical event described for the first time in 1880 by the neurologist and psychiatrist Jules Cotard and characterized by negation delusions (or nihilists). Immortality and hypochondriac delusions are also typical. Nowadays, it is known that CS can be associated with many neuropsychiatric conditions. In this article, we describe the case of a patient that believed not having more organs and having the body deformed and whose CS was associated with a bigger depressive disorder. Although the electroconvulsive therapy is the most described treatment modality in the literature, the reported case had therapeutic success with association of imipramine and risperidone.

## 1. Introduction

Cotard's Syndrome (CS) is a rare clinical event, characterized by negation delusion (or nihilist), generally regarding the body (frequently the patient believes that he or she does not have one or more organs) or regarding the existence (the individual judges that himself or everybody in the world is dead or reduced to nothing, being able to judge himself a zombie), but also concerning concepts/conditions [1] (such as a CS case described in which a woman was sure about not being pregnant, despite of obvious evidences [2]). Besides that, other typical delusions are immortality delusion (the individual believes that nothing more can kill him, in this case because he is already dead) [3] and hypochondriac delusions (in which the patient judges to be suffering of a very serious or incurable disease or having the body deformed) [1, 4].

This condition was described with details in 1880 by the Parisian neurologist and psychiatrist Jules Cotard [5]. He described the clinical condition of a 43- year-old woman who affirms not to have many organs, but only a body in decomposition. Initially, Cotard formulated that the condition would be a new kind of depression characterized by anxious melancholy, condemnation of ideas, insensitivity to pain, negation delusion of the organs, and immortality delusion. Sometime later, he created the term negation delusion to denominate the syndrome [6, 7].

Nowadays, it is known that this syndrome may be secondary to many conditions such as psychotic depression [8–11], schizophrenia [3], bipolar disorder [12, 13], dementia [14], cerebral neoplasm [15], cerebral hemorrhage [16], and use of psychoactive substances [17], among other neuropsychiatric conditions. Besides that, associations of CS with other syndromes are described, such as catatonic syndrome [18], malignant neuroleptic syndrome [19], lycanthropy [20], hydrophobia [21], and capgras syndrome [22, 23].

Although rare, CS involves big suicide risk and, because of that, it is recommended to institute treatment as early as possible [7]. Thus, it is necessary that the clinicians be aware to the many possibilities of manifestation of this syndrome and, because of that, we describe in this article a CS case with negation delusions of organs and hypochondriac.

## 2. Case Presentation

Patient, a 31-year-old male, was examined in the Sector of Psychiatric Emergency of a Psychiatric Hospital of Brazil presenting insomnia, feed refusing, soliloquies, and hanging suicide attempt. He suspended his previous psychiatric medicines by himself. There were no other clinical changes. At the mental exam, he presented poor hygiene, disheveled hair, unshaven, and passive posture, he was self-referential, alert, with hypoprosexia, oriented, he had seriously depressed humor, intense psychomotor retardment, and very slowly thinking and with many blocks. He reported that his organs were destroyed, that he was hollow inside, and that he was a rotten and smelly flesh (negation delusion regarding the body, including the organs). He referred that his body was deformed and that his face was full of holes (hypochondriac delusion), including trying to show to the examiner doctor. He referred will to die. He said that he was paying for what he had done wrong in the past (guilty delusion) and that he was not worthy feeling happiness, besides to be sure that he would never see the sun light again (condemnation delusion). The patient was hospitalized. It was diagnosed CS secondary to serious depressive disorder with psychotic symptoms. As the service did not dispose of an electroconvulsive therapy machine, he was prescribed imipramine 150 mg/day associated with risperidone 6 mg/day. The patient was released from the hospital, about 60 days later, asymptomatic.

## 3. Discussion

We described a CS case secondary to a serious depression with psychotic symptoms. The main delusion involved in the syndrome, the negation one, stands out in the condition. Besides it, hypochondriac delusion as the way described by Cotard (an interpretation of pathological sensations) [1] was present, as well as guilty and condemnation delusions congruent with the depressed humor. Among 100 cases described in the literature analyzed by Berrios and Luque [1], 89 had depressed humor and the most commonly found delusion was the nihilist related to the body (86 cases). Besides this delusion, another characteristic of CS that was reported in our case, the hypochondriac, was present in 58 patients. The delusions that are common in CS and were present in our report, despite not defining a syndrome, were found in 63 reports (guilty delusion) and in 35 (condemnation delusion).

CS is a rare condition; therefore it is not clear which treatment should be considered as the first choice. The electroconvulsive therapy (ECT) has been the preferred treatment form for CS in several case reports [7, 10, 14, 19]. This preference is possibly justified by the fact that CS is a serious psychotic syndrome, generally associated with an equally serious humor situation and with high suicide risk. However, especially in Brazil, the ECT is not always an easy availability option because its use is not financed by the public service and the stigma is still an important limiting factor. The medication treatment is also described as successful [2, 3, 8, 11, 18, 22] and, in these cases, frequently with preference for using antipsychotics in association with antidepressants [8, 11] as exemplified in this report. Anyway, the treatment should be directed to the basis cause.

In 1968, Saavedra classified CS as genuine CS, which occurred only in depressive states, and the pseudo-CS [7]. However, it was only in 1995 that appeared a classification based on evidence. Through retrospective analysis of 100 case reports in the literature, Berrios and Luque [1] subdivided the syndrome into three subtypes. The first type, denominated psychotic depression, includes patients with melancholia and few nihilist delusions. The second, called CS type I, includes conditions that course with nihilist and hypochondriac delusions in absence of humor changes. And the third, denominated CS type II, includes heterogeneous conditions which course with anxiety, depression, immortality delusion, nihilist delusion, and/or auditory hallucination. Nowadays, CS is not classified as an isolated disorder in the 5th edition of Diagnostic and Statistic Manual of Mental Disorders (DSM-5) neither in the International Classification of Diseases (ICD-10). At DSM-5, nihilist delusion is classified as delusion congruent with the humor inserted in a grave depressive episode with psychotic symptoms [24, 25]. Our report can be classified as genuine CS, once it is associated with a depressive disorder, as well as CS type II for presenting depression and nihilist delusion. At DSM-5, however, it would be classified only as a more severe depressive disorder with psychotic characteristics congruent with the humor.

From the neurobiological point of view, there are some reports with PET scan studies which suggest a hypometabolism of the frontal-parietal-temporal cortex associative circuit [6, 26, 27] and a hypermetabolism in the basal ganglia [26, 27] and cerebellum [26]. However, it was not possible to proceed neuroimaging studies for the reported patient due to the lack of availability of these examinations in our region through the public health system.

## 4. Conclusion

Although rare and described more than 120 years ago, CS is still a present phenomenon in the psychiatric clinics all over the world with clinical characteristics similar to those initially observed by Jules Cotard. The case described here has a function of calling the clinicians' attention. Besides that, it shows that, even in places with lack of technologic resources (public hospital without neuroimaging and without ECT service), it is possible to recognize the situation by the clinical presentation and obtain therapeutic success with older psychotropic drugs.
